# Warmth and competence stereotypes about immigrant groups in Germany

**DOI:** 10.1371/journal.pone.0223103

**Published:** 2019-09-27

**Authors:** Laura Froehlich, Isabel Schulte

**Affiliations:** Faculty of Psychology, University of Hagen, Hagen, Germany; York University, CANADA

## Abstract

Germany is ethnically diverse and the social climate is more or less welcoming for different immigrant groups. The social climate can be described by stereotypes of members of the receiving society about immigrant groups, which in turn shape receiving-society members’ behavioral tendencies of support or discrimination. We investigated warmth and competence stereotypes about 17 immigrant groups in Germany. Results showed four clusters of immigrant groups in the two-dimensional space of warmth and competence. Groups who immigrated comparatively recently and from regions of conflict (e.g., the Balkans, Northern Africa) were stereotyped most negatively (moderate warmth, low competence). Across groups, path analysis investigated the socio-structural relations proposed by the stereotype content model and the BIAS map for immigrant groups in the German context. In a pre-registered model all hypothesized paths were significant but model fit was not good. Therefore, an exploratory model included additional paths as well as intercorrelations between exogenous variables and error terms. The modified model showed good fit and partly replicated the relations proposed by the BIAS map. Threat predicted warmth, whereas status predicted competence. Warmth predicted active behavioral tendencies and competence predicted passive behavioral tendencies. Additional paths from status to warmth, threat to competence, as well as from warmth to passive behavioral tendencies and competence to active behavioral tendencies were also significant. Thus, findings support receiving-society members’ active role in the process of integrating immigrant groups into German society. Based on the results, social-psychological approaches to foster immigrant integration are discussed.

## Introduction

Many European countries have ethnically heterogeneous populations and have received large numbers of immigrants during the last decades. Germany, the United Kingdom, France, and Spain are among the top ten countries with the highest proportions of immigrants in the world [1]. Following the United States of America and Saudi Arabia, Germany has the third-highest proportion of immigrants worldwide [1]: Twenty-three percent of Germans have a migration background, that is, they or their parents were born in another country [2]. Germany’s ethnically heterogeneous population is a challenge for societal integration and cohesion [3]. Immigrants in Germany have diverse ethnic backgrounds and migration histories. For example, former ‘guest workers’ who have mainly emigrated from Turkey, former Yugoslavia and Greece have often lived in Germany for several decades [4]. However, since 2014 there has been a recent increase of immigration to Germany, mainly from regions of conflict, e.g., Syria, Afghanistan, and the Balkans [5].

It is likely that various immigrant groups face different (un)welcoming social climates in Germany. From a social-psychological perspective, these social climates can be operationalized by the attitudes of members of the receiving society towards immigrant groups [6–9]. Research showed that receiving-society members’ views of immigrants have consequences for the integration process. Unwelcoming social climates (i.e., negative views) are related to higher endorsement of cultural maintenance by immigrants [6] as well as an emphasis on immigrants’ identities related to their origin culture [7], which can lead to an adoption of the acculturation orientation of separation and corresponding integration difficulties. Furthermore, unwelcoming social climates are related to negative behavioral tendencies of discrimination and exclusion towards immigrants by members of the receiving society [10]. In contrast, welcoming climates (i.e., positive views) are related to efforts of members of the receiving society to integrate immigrants and thereby shape a society in which all groups can equally participate [8, 9]. An investigation of social climates for different immigrant groups in Germany is thus relevant for achieving societal integration of all ethnic groups, as it provides knowledge about which immigrant groups are able to integrate relatively well and which groups are facing integration difficulties. To increase the understanding of the social climate for immigrants in Germany, the current research focuses on socially shared stereotypes about different immigrant groups. Stereotypes are “beliefs and associations that link a whole group of people with certain traits or characteristics” [11]. Stereotypes stem from socio-structural relations between groups and predict in how far members of the receiving society tend to behave towards immigrant groups positively (i.e., support and help) or negatively (i.e., discrimination and exclusion) [12, 13]. Detailed knowledge of receiving-society members’ stereotypes about various immigrant groups in Germany is thus relevant for immigrant integration.

### Stereotypes about immigrants in terms of the stereotype content model

An influential social-psychological model of stereotypes is the stereotype content model [14, 15], which posits that social groups are stereotyped on two basic dimensions: Warmth and competence. *Warmth* refers to likability and friendliness, and is predicted by perceptions of outgroups’ intentions toward the ingroup. These intentions are reflected by competition or realistic threat (i.e., perceived threat to the ingroup’s political and economic power) as well as symbolic threat (i.e., perceived threat to the ingroup’s worldviews in terms of attitudes and values) [16, 17]. In turn, *competence* refers to capability and confidence and is predicted by perceptions of status, reflecting the outgroups’ abilities to put their intentions into practice (i.e., in how far they have high social status and corresponding resources like wealth, education and employment) [10, 17].

Cross-cultural studies showed that the basic dimensions of stereotyping are universal, but stereotypes also differ depending on the socio-cultural context. For example, the group composition within a society as well as intergroup relations or cultural and economic factors can shape stereotypes [18, 19]. In general, immigrants are often stereotyped negatively as cold and incompetent [12]. However, not all immigrant groups are stereotyped alike [12, 13, 20]. Rather, stereotypes about immigrant groups vary along warmth and competence and for many groups, stereotypes are likely to be ambivalent (i.e., high on one dimension and low on the other) [12, 21–23].

In the German context, three studies investigated the stereotype content model: Eckes [24] investigated stereotypes about 41 social groups, including gender-related subgroups (e.g., feminist, career woman, macho, manager; Study 1) and a broader variety of social groups (e.g., senior citizens, immigrants, Turks, homosexuals; Study 2) with participants from Eastern Germany. Immigrants were stereotyped as cold and incompetent, whereas Turks–the largest group of immigrants in Germany–were stereotyped as moderately warm and competent. Asbrock [21] investigated the validity of the stereotype content model for the Western German context with 27 social groups (e.g., senior citizens, unemployed, rich people, gay men), among them three immigrant groups (i.e., Asians, Turks, foreigners). Turks and foreigners were stereotyped as moderately warm and competent, whereas Asians were stereotyped as warm and competent. These two studies showed that the two-dimensional structure of the stereotype content model could be replicated in the German context when investigating a broad variety of social groups, but only a very limited selection of immigrant groups was included. However, the fact that Turks and Asians were stereotyped differently than the overall group of immigrants underscores that various immigrant groups are likely to receive different stereotypes in the German context.

Kotzur and colleagues [25] recently investigated stereotypes about 11 refugee groups (e.g., Syrian refugees, Muslim refugees, refugees from the Balkans) and five reference groups derived from previous research (e.g., rich people, elderly people) in Germany and showed that stereotypes about refugee subgroups could be mapped on warmth and competence and varied according to region of origin, flight motive, and religious affiliation of refugee groups. In contrast to these previous works and to close a gap in the literature about stereotypes about immigrant groups in Germany, the current study focuses on a broader selection of immigrant groups in Germany based on their country of origin. In light of the recent increase in immigration to Germany and shifts from labor migration to flight from regions of conflict, a current detailed investigation of stereotypes about major immigrant groups–including traditional labor immigrants *as well as* recently immigrated groups–is needed in order to capture the social climate for immigrants in Germany.

With the present study, we intended to contribute to the literature by investigating receiving-society members’ stereotypes about 17 immigrant groups from different countries of origin. In line with research from other national contexts [12, 13, 20], we expected stereotypes to vary on warmth and competence and some immigrant groups to receive ambivalent stereotypes (i.e., higher warmth than competence or vice versa). This should be the case because immigrant groups vary in status (e.g., wealth, education, and employment), which predicts competence, as well as perceived threat (e.g., cultural and religious values and traditions) which predicts warmth [13, 26]. Furthermore, Kotzur et al. (2019) showed that stereotypes about refugee subgroups in Germany were related to their migration status and religious affiliation. Applied to the more general context of immigration, we thus expect traditional labor migrants to be stereotyped more positively on warmth and competence than refugees [25, 26]. Furthermore, we expect immigrants from predominantly Muslim countries to be stereotyped more negatively on warmth and competence than immigrants from countries with predominantly non-Muslim populations [22, 25]. It should be noted that as the paper by Kotzur et al. (2019) was published after the current study was pre-registered and conducted, these additional expectations were generated post-hoc and should thus be substantiated by further research.

Furthermore, intergroup contact has been shown to predict intergroup attitudes [27–29]. Whereas positive contact (i.e., positive interactions with outgroup members) has been consistently shown to reduce intergroup prejudice [27], there is also the possibility of negative contact (i.e., negative interactions with outgroup members; e.g., being discriminated, insulted or mistreated). Negative contact can in turn negatively impact intergroup attitudes and relations [30]. In the context of immigration, members of the receiving society likely have varying opportunities for positive and negative contact with different immigrant groups. Therefore, we control for positive and negative contact in an exploratory analysis.

### Stereotypes predict receiving-society members’ behavioral tendencies towards immigrants

Stereotypes are relevant for the integration of immigrants into multicultural European societies like Germany, as they predict receiving-society members’ behavioral tendencies of support or discrimination towards immigrant groups [10, 31, 32]. The Behaviors of Intergroup Affect and Stereotypes (BIAS) map [10] describes how stereotypes and intergroup emotions predict behaviors towards outgroup members. Behaviors are described in terms of intensity (active vs. passive) and valence (facilitation vs. harm). Because warmth is directly related to the perceived intent of the outgroup towards the ingroup, it is assumed to have a primary role for intergroup behavioral tendencies [10]. Thus, warmth predicts active behavioral tendencies, whereas competence predicts passive behavioral tendencies. Warm groups are actively facilitated (e.g., helped or defended), whereas cold groups are actively harmed (e.g., discriminated). In turn, competent groups are passively facilitated (e.g., tolerated), whereas incompetent groups are passively harmed (e.g., ignored or excluded) [10, 32].

Because members of the receiving society play an active role in the integration process [33], their endorsement of positive stereotypes can thus facilitate immigrant integration, whereas endorsement of negative stereotypes can hinder integration. In case of ambivalent stereotypes, conflicting behavioral tendencies are activated: High warmth and low competence predict active facilitation and passive harm, whereas high competence and low warmth predict passive facilitation and active harm. Addressing these conflicting predictions for behavioral tendencies in case of ambivalent stereotypes, Becker and Asbrock [32] showed that the salience of the stereotype dimension predicts which behavioral tendency is more likely to be activated. If the dimension that is stereotyped low is salient, negative behavioral tendencies (i.e., active/ passive harm) are likely. In turn, if the dimension that is stereotyped high is salient, positive behavioral tendencies (i.e., active/ passive facilitation) are likely. Thus, detailed knowledge about which immigrant groups are stereotyped ambivalently in the German context and which dimensions are stereotyped to be high/low for the particular groups is relevant for predicting under which conditions the respective groups are treated positively or negatively by members of the receiving society.

In addition to mapping stereotypes about different immigrant groups in the two-dimensional space of warmth and competence, the second major goal of the present research was to investigate how the socio-structural variables of status and threat predict warmth and competence stereotypes about immigrant groups in Germany as well as the behavioral tendencies of members of the receiving society. The main aim of this investigation was to replicate socio-structural relations proposed by the stereotype content model and the BIAS map [10] that constitute the different (un)welcoming social climates for immigrant groups in the German context. Across groups, we expected threat to predict warmth, and status to predict competence. In turn, warmth was expected to predict active behavioral tendencies and competence to predict passive behavioral tendencies. In line with recent methodological critique of traditional analysis strategies to test predictions of the stereotype content model by Kotzur and colleagues [25, 28], and to extend previous research which often investigated each of the outcome variables in separate regression models (but see Kotzur, Schäfer, and Wagner (2018) for a recent counterexample), we use path analysis to investigate the expected relations in a joint path model.

## Methods

### Ethics statement

The procedures of the studies were in compliance with the Ethical Principle of the WMA Declaration of Helsinki. No special permission is required in Germany for the conduction of studies based on anonymous and confidential questionnaires that are not expected to entail any lasting harms or risks for the participants, so IRB approval was not necessary. All procedures were performed in full accordance with the ethical guidelines of the DGPs (Deutsche Gesellschaft für Psychologie–German Society for Psychology) [34].

### Pilot study: Sampling of relevant immigrant groups

Thirty participants (age: *M* = 36.73 years, *SD* = 17.50, 14 female) listed up to 20 immigrant groups living in Germany and were instructed to consider all regions of Germany for their answers. Participants were Germans born in Germany with varying educational degrees (Hauptschulabschluss (basic secondary education certificate, 9 years of schooling): *n* = 2; Realschulabschluss (intermediate secondary education certificate, 10 years of schooling): *n* = 8; Fachhochschulreife (advanced technical college certificate, 11/12 years of schooling): *n* = 4; Abitur (high school certificate, 12/13 years of schooling: *n* = 8); university degree (*n* = 8); and were recruited via mailing lists and personal communication. In total, 38 immigrant groups were listed. Groups listed by at least 20% of the participants were selected [21]. The following immigrant groups were used for the main study (listed according to frequency): Immigrants from Turkey (97%), Russia (93%), Syria (80%), Italy (77%), Poland (73%), African countries (47%), Romania (40%), Greece (37%), Afghanistan (33%), Albania (27%), China (27%), Bulgaria (23%), Pakistan (20%), and Arabic countries (20%). Furthermore, immigrants from Morocco, Tunisia, and Egypt (all 7%) were included due to frequent media coverage about immigrants from “North Africa” in 2016 [35].

### Main study: Participants and procedure

The main study consisted of an online questionnaire, which was completed by *N* = 217 participants. Participants were recruited through the virtual lab of the University of Hagen, a distance learning university that has a majority of non-traditional students (i.e., on average older than traditional students and often working full time while studying) and mailing lists (e.g., “psychological studies for everyone”, “university message board”). Questionnaire completion took approximately 30 minutes. To exclude speeders, we removed participants who took less than 10 minutes to respond (*n* = 17; i.e., less than 30% of the median response time and less than the estimated reading speed of 300 ms per word) [36, 37]. The final sample consisted of *N* = 200 participants (age: *M* = 35.33 years, *SD* = 11.69, 150 female, 48 male, 1 trans-/intersexual, 1 missing). Seventy-seven percent of the participants were enrolled at the University of Hagen and received course credit for participation; the non-student participants did not receive compensation. Six percent of participants had a non-German nationality, predominantly these participants came from the neighboring countries of Austria and the Netherlands. The percentage of missing values ranged from zero to 7% (overall missing values: 1.4%). Data were missing at random (Little’s test: χ²(2298) = 2066.27, *p* > .99) and thus we used pairwise deletion or estimation using the FIML algorithm. Data, materials (in German and English language), scripts and results are available on the Open Science Framework: https://osf.io/ty5jx/

Participants were informed that data were treated anonymous and would be used for scientific purposes only, and gave written consent following the ethical code of conduct of the American Psychological Association [38]. Participants were assured that their participation was voluntary, that they could cancel participation at any time and that data were anonymized. To avoid fatigue, participants were presented with five groups that were randomly selected out of the 17 immigrant groups. To reflect stereotypical perception of the ethnic majority group, Germans were also rated on warmth and competence by all participants. They were instructed not to indicate their personal ratings of the groups, but the general perception of these groups by Germans to reduce social desirability and to assess culturally and socially shared stereotypes [13, 14, 17, 21].

The pre-registration of the study is available at https://aspredicted.org/np3xh.pdf. A sample size of *N* = 300 was pre-registered in order to be able to conduct complex statistical analyses (e.g., path modeling). Data were collected as part of a student thesis of the second author in the fixed period of 3 months (January—April 2017). Because the pre-registered sample size could not be reached in that fixed period of time, we conducted a post-hoc power analysis to investigate the test power of the current study given the empirical effect size and the achieved sample size. Results indicated that test power was sufficiently high: For the highest level of analysis (groups), test power for paired-samples *t*-tests for mean comparisons of warmth and competence stereotypes with a mean sample size of *N* = 66 per group and a mean empirical effect size of *d* = .51 was 1 - β = .82.

### Materials

Internal consistencies of scales are indicated by Spearman’s Rho (2 items) or Cronbach’s Alphas (> 2 items). The groups were rated on perceived *status* (3 items: “To what extent is [group] considered by most people to have…”: prestigious jobs, economic success, a good education; .80 > α > .94) [13]; perceived *threat* (2 items: realistic threat: “If resources go to [group], this takes resources away from the rest of society” and symbolic threat: “The values and beliefs of [group] are not compatible with the beliefs and values of most Germans”, .29 > *r* > .93, all *p*s < .05) [17]; *warmth* (4 items, “[Group] are warm, likable, good-natured, friendly;. 89 > α > .97); and *competence* (4 items; competent, independent, competitive, capable; .86 > α > .95) [17, 21]. Furthermore, we assessed *behavioral tendencies* towards the immigrant groups with the items “Germans tend to … [group]”: active facilitation (i.e., help, protect; .61 > *r* > .89, all *p*s < .001), active harm (i.e., fight, attack; .51 > *r* > .93, all *p*s < .001), passive facilitation (i.e., cooperate with, associate with; .44 > *r* > .90, all *p*s < .01), and passive harm (i.e., exclude, demean; .35 > *r* > .79, all *p*s < .01) [10]. Answers were indicated on a scale from 1 = *not at all* to 5 = *completely*. Furthermore, we assessed positive contact (“How often do you have positive contact to immigrants?”) as well as negative contact (“How often do you have negative contact to immigrants?” measured on a scale from 1 = *never* to 7 = *very often*.

Finally, participants provided demographics including gender, age, and migration background. We also measured majority integration efforts as a more detailed measure of active facilitation behavioral tendencies towards immigrants with 21 items [8]. To reduce questionnaire length, the scale was adapted: Items measuring integration efforts on a general level (i.e., no reference to specific groups) were rated only once (10 items). Items directly referring to specific groups were assessed for each group (11 items). Unfortunately, this resulted in high variation of internal scale consistencies (.10 > α > .80) and insufficient consistencies for some groups, thus majority integration effort items were excluded from the analyses.

## Results

### Stereotype content

**Fig 1:**
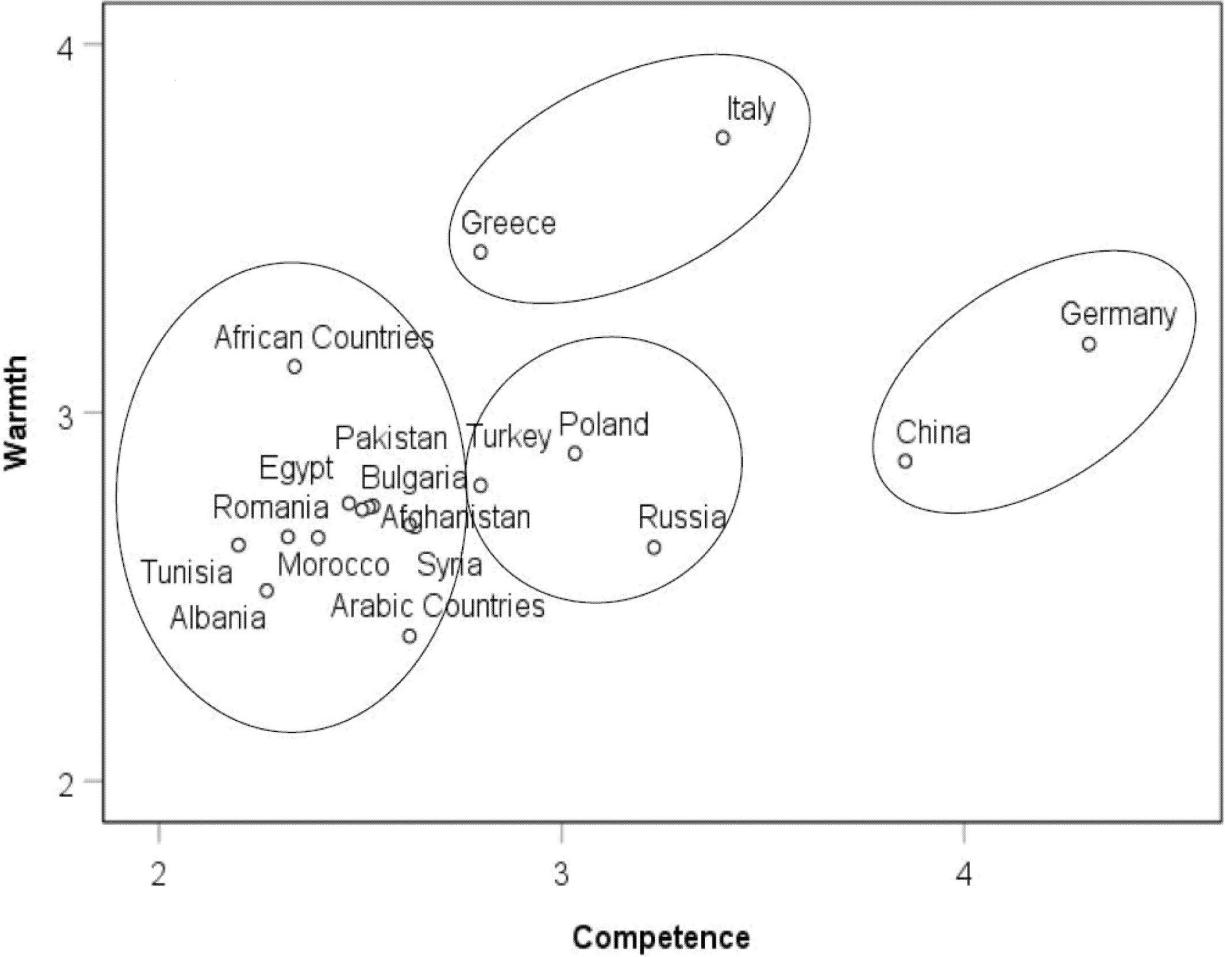
Warmth by competence space mapping the clusters of immigrant groups (*N* = 18).

To investigate group differences in warmth and competence ratings, data were aggregated to the group level (*N* = 18, including the 17 immigrant groups as well as the group of Germans) [21] and a hierarchical cluster analysis (Ward method) was employed to determine the number of clusters. A subsequent k-means cluster analysis determined the optimal allocation of groups in the clusters. A 4-cluster solution was chosen (Fig 1) based on the agglomeration schedule. Mean differences of warmth and competence within groups and clusters were tested with paired-samples *t*-tests (Table 1). Cluster 1 (high competence/ moderate warmth) consisted of Germans and immigrants from China. Cluster 2 (moderate competence/ moderate warmth) encompassed immigrants from Poland, Russia and Turkey. Groups that recently migrated to Germany were grouped in Cluster 3 (low competence/ moderate warmth): Immigrants from Afghanistan, Syria, Tunisia, Egypt, Morocco, African Countries, Arabic Countries, Bulgaria, Romania, Albania, and Pakistan. Cluster 4 (moderate competence/ high warmth) encompassed immigrants from Italy and Greece. Results of the cluster analyses remained similar when the superordinate groups “Arabic Countries” and “African Countries” were excluded. Cluster 3 was rated significantly higher in warmth than competence (Table 1). With regard to the specific immigrant groups, immigrants from China and Russia were rated higher in competence than warmth, whereas immigrants from Tunisia, Egypt, Morocco, African Countries, Romania, Albania, Italy, and Greece were rated higher in warmth than competence. For all other groups, warmth and competence ratings did not differ significantly.

**Table 1:** Warmth and competence means of cluster centers and groups.

		Warmth	Competence					
Cluster	Groups	*M (SD)*	*M (SD)*	Diff [95% CI]	*t*	*df*	*p*	*d*
1		3.03 (0.22)	4.08 (0.32)	1.05 [0.17; 1.94]	15.20	1	.042	
	Germany	3.19 (0.73)	4.31 (0.69)	1.12 [1.01; 1.24]	19.50	197	< .001*	1.38
	China	2.87 (0.73)	3.85 (0.76)	0.99 [0.78; 1.19]	9.71	56	< .001*	1.28
2		2.81 (0.14)	2.99 (0.17)	0.18 [-0.80; 0.44]	1.28	2	.330	
	Poland	2.90 (0.96)	3.03 (0.89)	0.12 [-0.16; 0.41]	0.88	46	.384	0.13
	Russia	2.63 (0.83)	3.23 (0.80)	0.60 [0.38; 0.81]	5.54	63	< .001*	0.70
	Turkey	2.80 (0.73)	2.80 (0.75)	0.01 [-0.16; 0.17]	0.05	61	.961	0.02
3		2.70 (0.16)	2.44 (0.14)	0.26 [0.11; 0.40]	3.88	10	.003*	
	Afghanistan	2.75 (0.86)	2.53 (0.81)	0.21 [0.01; 0.42]	2.10	62	.040	0.27
	Syria	2.70 (0.84)	2.62 (0.85)	0.07 [-0.12; 0.27]	0.75	54	.456	0.11
	Tunisia	2.64 (0.94)	2.20 (0.82)	0.44 [0.28; 0.61]	5.42	70	< .001*	0.64
	Egypt	2.75 (0.72)	2.47 (0.73)	0.28 [0.09; 0.47]	2.93	66	.005*	0.36
	Morocco	2.66 (0.88)	2.32 (0.85)	0.34 [0.13; 0.55]	3.28	42	.002*	0.50
	Afr. Countr.	3.12 (0.75)	2.34 (0.68)	0.78 [0.56; 1.00]	7.00	54	< .001*	0.95
	Arab. Countr.	2.39 (0.84)	2.62 (0.80)	0.23 [0.01; 0.47]	1.94	53	.058	0.27
	Bulgaria	2.74 (0.88)	2.52 (0.75)	0.22 [0.02; 0.43]	2.17	59	.034	0.28
	Romania	2.65 (0.67)	2.39 (0.72)	0.27 [0.08; 0.45]	2.90	57	.005*	0.39
	Albania	2.52 (0.84)	2.27 (0.81)	0.25 [0.07; 0.43]	2.78	58	.007*	0.36
	Pakistan	2.77 (0.89)	2.53 (0.95)	0.24 [0.05; 0.42]	2.51	59	.015	0.32
4		3.54 (0.16)	3.06 (0.39)	0.48 [-1.08; 2.05]	3.91	1	.159	
	Italy	3.75 (0.65)	3.40 (0.59)	0.35 [0.20; 0.49]	4.86	60	< .001*	0.63
	Greece	3.42 (0.84)	2.80 (0.85)	0.62 [0.38; 0.87]	5.03	57	< .001*	0.66

* = below significance level (Bonferroni-adjusted *p* = .008 for comparisons of immigrant groups, *p* = .013 for comparisons of cluster centers).

### A path model of threat and status, stereotypes, and behavioral tendencies

**Fig 2:**
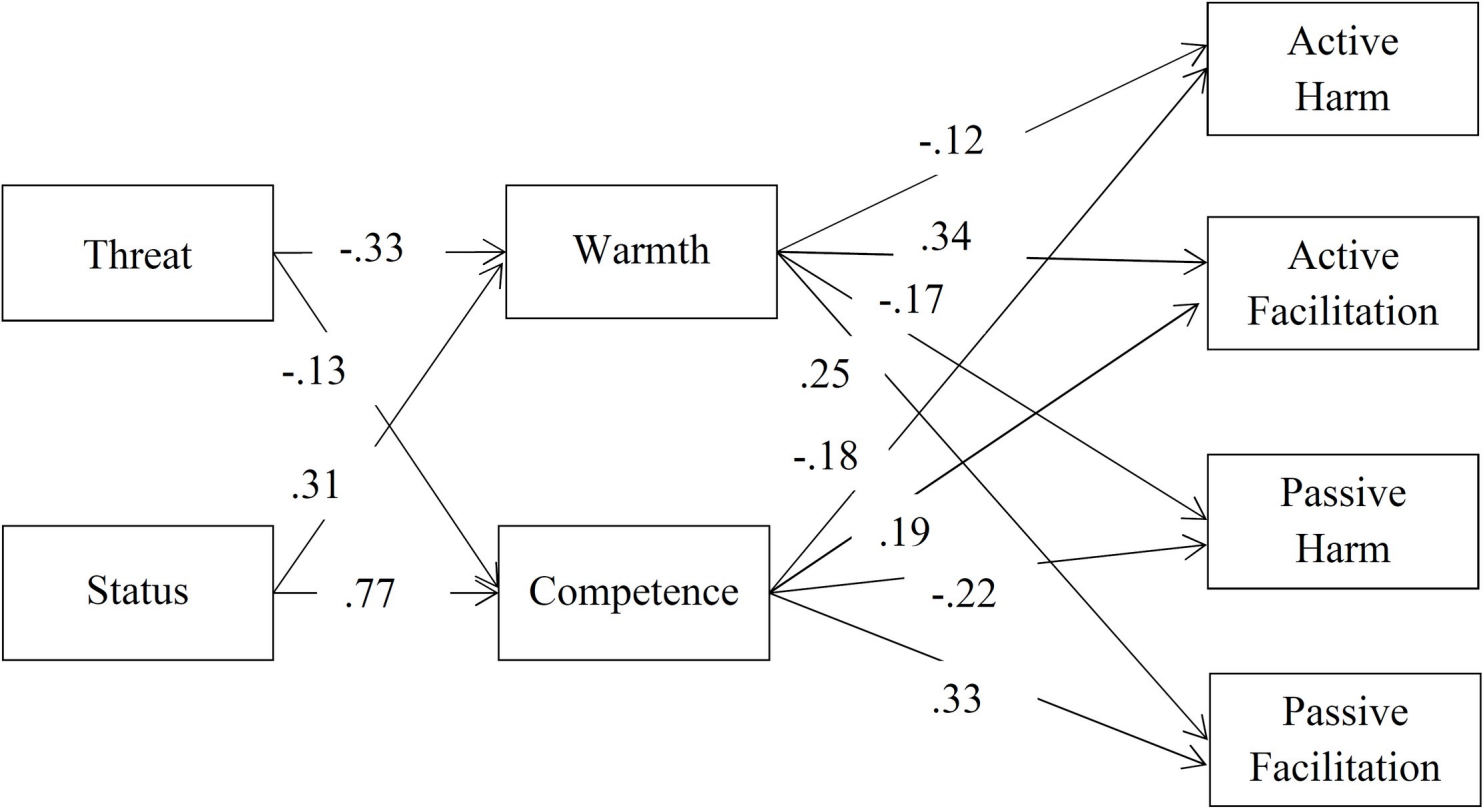
Results of modified path model.

To investigate whether threat and status predict stereotypes, which in turn predict behavioral tendencies across the 17 immigrant groups, we restructured the data into long format (i.e., the measurement level). The two items measuring symbolic and realistic threat were highly correlated and were thus combined to one scale. We computed a path model in Mplus [39] accounting for the complex data structure (type = complex; measurements nested within persons). In accordance with the pre-registration, we computed a model in which threat predicted warmth, status predicted competence, warmth predicted active behavioral tendencies, and competence predicted passive behavioral tendencies. The exogenous variables of threat and status were allowed to correlate. Intercorrelations of error terms were fixed to zero. Although all hypothesized paths were significant, model fit was not good (RMSEA = .21, CFI = .49, TLI = .34, SRMR = .21). Therefore, based on modification indices we computed an exploratory model including additional paths from threat to competence, status to warmth, warmth to passive behavioral tendencies and competence to active behavioral tendencies. Furthermore, error terms between warmth and competence as well as between behavioral tendencies were allowed to correlate. Model fit of the modified model was good (RMSEA = .08, CFI = .97, TLI = .91, SRMR = .04). All additional paths were significant, and all original paths remained significant (all *p*s < .027). Results of the modified model are shown in Table 2 and Fig 2.

**Table 2:** Estimated effects of the modified path model.

Outcomes	β (SE)	[LLCI; ULCI]	*p*	β (SE)	[LLCI; ULCI]	*p*
	*Threat*			*Status*		
Warmth	-.33 (0.04)	[-.41; -.25]	< .001	.31 (0.04)	[.23; .38]	< .001
Competence	-.13 (0.02)	[-.18; -.09]	< .001	.77 (0.02)	[.73; .81]	< .001
	*Warmth*			*Competence*		
Active Harm	-.12 (0.05)	[-.22; -.02]	.024	-.18 (0.05)	[-.27; -.08]	< .001
Active Facilitation	.34 (0.05)	[.24; .43]	< .001	.19 (0.05)	[.09; .28]	< .001
Passive Harm	-.17 (0.05)	[-.27; -.07]	.001	-.22 (0.05)	[-.32; -.11]	< .001
Passive Facilitation	.25 (0.05)	[.17; .34]	< .001	.33 (0.05)	[.24; .43]	< .001

*N* = 200 participants (L2), *N* = 1000 observations (L1). Confidence intervals are displayed at the 95% level.

As expected and replicating predictions by the BIAS map, threat negatively predicted warmth and status positively predicted competence. In turn, warmth negatively predicted active harm and positively predicted active facilitation. Competence negatively predicted passive harm and positively predicted passive facilitation. Furthermore, all additional exploratory paths were also significant. Threat negatively predicted competence, whereas status positively predicted warmth. In turn, warmth negatively predicted passive harm and positively predicted passive facilitation. Competence negatively predicted active harm and positively predicted active facilitation. Threat and Status were moderately and negatively correlated (*r* = -.38, *p* < .001). Correlations of residuals were also significant (all *p*s < .001). The model explained the following amounts of variance in the dependent variables: warmth (29%), competence (71%), active harm (7%), active facilitation (21%), passive harm (11%) and passive facilitation (26%). Models excluding participants with non-German nationality (*n* = 11) or controlling for positive and negative contact with immigrant groups showed similar results.

To test whether the exploratory paths and the pre-registered paths from warmth and competence stereotypes to behavioral tendencies differed in magnitude–and thus to investigate differences in contributions of the newly added paths and the paths included in the traditional formulation of the stereotype content model to the prediction of behavioral tendencies–in a series of constrained models both paths (from warmth and competence to each behavioral tendency) were restricted to be equal. Model fit of the constrained and unconstrained models was compared using χ² difference tests. For active facilitation the constrained model fit to the data significantly worse than the unconstrained model (Δχ²(1) = 4.04, *p* = .044). This means that active facilitation was predicted by warmth more strongly than by competence. For all other behavioral tendencies model fit of the constrained models was not significantly worse than that of the unconstrained models (0.23 < Δχ² < 1.20; .273 < *p* < .631). Thus, regression weights of warmth and competence for the prediction of active harm, passive facilitation and passive harm did not significantly differ from each other.

## Discussion

Germany’s population is highly and increasingly multicultural, and the integration of immigrants remains a societal challenge [3]. Immigrant integration depends on the social climate for different ethnic groups, which can be described by how members of the receiving society view immigrants in terms of the stereotype content model and the BIAS map [10, 14]. The current research provides a systematic investigation of receiving-society members’ stereotypes about various immigrant groups in Germany and related behavioral tendencies.

### Stereotypes about immigrant groups in Germany

In accordance with international studies [12, 13, 20], immigrant groups in Germany were stereotyped differently along the dimensions of warmth and competence. Thus, the high ethno-cultural diversity of immigrant groups in Germany is reflected by socially shared stereotypes about these groups [40]. In accordance with the stereotype content model’s prediction that stereotypes about social groups vary in terms of warmth and competence, four clusters emerged: High competence/ moderate warmth (Germans and immigrants from China); moderate competence/moderate warmth (immigrants from Poland, Russia, and Turkey); low competence/ moderate warmth (immigrants from Afghanistan, Syria, Tunisia, Egypt, Morocco, African Countries, Arabic Countries, Bulgaria, Romania, Albania, and Pakistan); and moderate competence/ high warmth (immigrants from Italy and Greece).

In line with the investigation of stereotypes about refugee subgroups by Kotzur et al. (2019), an exploratory investigation showed that factors driving the social perception of different immigrant groups in Germany might be their migration status (labor immigrants vs. refugees) and their religious affiliation (non-Muslim vs. Muslim) [25]. Traditional labor migrant groups like immigrants from Turkey, Italy, or Greece were stereotyped more positively than groups who have migrated to Germany comparatively recently and predominantly fled from regions of conflict [25]. Immigrants from the Balkans (i.e., Bulgaria, Romania, Albania) as well as Northern Africa and predominantly Muslim countries (i.e., Egypt, Tunisia, Morocco, Afghanistan, Syria, Pakistan) were perceived as moderate or low on both stereotype dimensions and were grouped together in a cluster of the most negative stereotypes [10]. Thus, these immigrant groups currently face the most unwelcoming social climate in Germany and will likely encounter most pronounced integration difficulties [41]. Taken together, labor migrant groups who are non-Muslim (with the exception of immigrants from Turkey) and have a longer history of residence in Germany were stereotyped more positively than immigrant groups who fled from regions of conflict, are Muslim, or have immigrated to Germany relatively recently.

Interestingly, in the current research groups who predominantly fled from regions of conflict were not stereotyped in the most negative way possible, as their ratings for warmth were higher than for competence. Due to relatively low perceptions of agency and control, it can be expected that refugees are stereotyped as incompetent. In turn, threat perceptions due to immigration by refugees (i.e., increased crime rates, competition on job and housing markets, as well as cultural influences by Islam) might lead to refugees being stereotyped as low on warmth as well [26]. However, a study investigating stereotypes about fled people (i.e., refugees or asylum seekers) in Germany showed that in line with results of the current research, refugees and asylum seekers were perceived as moderately warm rather than cold [26]. The authors discuss that fled people were associated with emotions of pity and admiration, which could also explain why in the current research immigrant groups from regions of conflict were stereotyped as incompetent, but moderately warm. Future research investigating stereotypes about different immigrant groups in Germany should thus include measures of intergroup emotions and take account of immigration status (e.g., refugees vs. labor migrants), religious affiliation (Muslim vs. non-Muslim) as well as migration history (e.g., time spent in the receiving society) as predictor variables.

### Socio-structural variables, stereotypes, and behavioral tendencies

The current research investigated the relations of stereotypes with the socio-structural variables of status and threat, as well as receiving-society members’ behavioral tendencies towards the immigrant groups in a joint path model. In line with recent methodological critique of traditional analysis strategies to test predictions of the stereotype content model [25, 28], the current research used path analysis to test the relations proposed by the BIAS map rather than conducting separate regression models for each of the outcome variables. This data-analytical technique allows for testing the fit of the model to the data. The pre-registered path model showed significant relations in the directions expected by the BIAS map [10], but the model did not fit the data well. This means that important relations between variables were missing in the pre-registered model. In order to increase the fit of the model to the data, an exploratory model included additional paths. Results of the modified model showed that across groups warmth and competence stereotypes were predicted by both threat and status. This means that the status-based group hierarchy as well as perceived threat of immigrants for resources and values were both relevant for perceptions of immigrant groups as friendly and capable. In turn, stereotypes on both dimensions were relevant for receiving-society members’ behavioral tendencies towards the immigrant groups.

The differential prediction of active/ passive behavioral tendencies by warmth and competence, respectively, was extended by significant paths from warmth to passive behavioral tendencies and from competence to active behavioral tendencies. As expected by the BIAS map, warmth was a stronger predictor of active facilitation than competence [10]. This result stresses the important role of the positive behavioral tendency of active facilitation, which is likely the most important contribution members of the receiving society can make for the integration of immigrants. All other behavioral tendencies were predicted equally by warmth and competence, contrary to the assumed primacy of warmth for the judgment of social groups [10].

The fact that the path model had to be modified to fit the data well indicates that socio-structural variables, stereotypes, and behavioral tendencies might be more strongly interrelated than originally suggested by the BIAS map–at least in the context of immigration in Germany. Previous research conducted with a broad variety of social groups has mostly supported the differential relationship of socio-structural variables and stereotypes, showing no relationships of status with warmth and competition with competence [14, 24, 42]; in other cases the diagonal correlations were not reported [18, 43]. One explanation for the unexpected significant diagonal paths in the current research could be the unique focus on immigrant groups. Whereas previous research investigated a broad selection of social groups, the current research focused specifically on immigrants. In the current heated debate in politics and the media about whether Germany can take in more immigrants or has reached its upper limit (“Obergrenze”), often concerns about status and realistic/ symbolic threat are mixed together (e.g., media articles portraying immigrants as a strain on the labor market, the welfare system, as well as the German cultural values and way of life). This might explain why participants perceived socio-structural variables, stereotypes and behavioral tendencies as closely related in the immigration topic. Further research should investigate the robustness of the exploratory paths while including emotional tendencies (i.e., admiration, contempt, envy, and pity) proposed by the BIAS map as mediators between stereotypes and behavioral tendencies in an extended path model or structural equation modeling framework.

### Practical implications

#### Receiving-society members’ contributions to immigrant integration

The societal integration of immigrants is not only shaped by the immigrant groups themselves, but also by members of the German majority group. The current research showed that socially shared stereotypes are related to receiving-society members’ behavioral tendencies towards immigrants. We investigated stereotypes and behavioral tendencies only across immigrant groups; however, it is an interesting question which immigrant groups are likely to be facilitated and which groups are likely to be harmed in the German context. Becker and Asbrock (2012) showed that when groups receive ambivalent stereotypes, behavioral tendencies are predicted by the more salient stereotype dimension [32]. Because none of the clusters in the current research clearly reflects univalent stereotypes (high competence/ high warmth or low competence/ low warmth), it is essential for receiving-society members’ behavioral tendencies towards immigrants which stereotypical dimension is salient in a given context or situation. Active facilitation is the most positive and direct tendency of support for immigrants, and it is more strongly predicted by warmth than competence. Thus, future research should investigate whether making salient the (positive) warmth stereotypes of almost all immigrant groups (except immigrants from China) influences competence stereotypes and behavioral tendencies towards these groups.

Because portrayals of immigrants in the media interact with receiving-society members’ attitudes to shape their reactions towards immigrants [35], how specific immigrant groups are portrayed in the media is relevant for their societal integration. Portrayals of immigrant groups as a threat to German resources and values will likely reduce receiving-society members’ tendencies towards active facilitation, whereas portrayals of immigrant groups as friendly and not threatening will likely increase active facilitation. Especially those immigrant groups who fled from regions of conflict and who are stereotyped as low in competence might benefit from more positive portrayals in the media on the warmth dimension.

#### Consequences of negative stereotypes for the integration of immigrants

The current study showed that when an immigrant group is stereotyped low on warmth or competence (or both), active and passive harmful behavioral tendencies are likely to increase. Active harm towards immigrants can be expressed via verbal or physical discrimination based on ethnic group membership. Experiences of discrimination can have adverse consequences for immigrants, for example lower quality of life and poorer health outcomes [44, 45]. In contrast, passive harm can be expressed via structural or institutional discrimination–a systematic reduction of immigrants’ opportunities for social and economic participation and integration [46]. Thus, stereotypes about immigrants can shape the social climate and thus the success of immigrant integration via majority members’ actions.

(Negative) stereotypes can also influence majority members’ causal attributions for the poor integration of specific immigrant groups. The attributional model of stereotypes [47] describes how negative stereotypes can lead to the attribution of negative outcomes (e.g., low educational success of immigrant groups) to characteristics that are internal to the immigrants (e.g., low ability or low effort). This tendency for internal attribution of negative outcomes for outgroups has been termed the *ultimate attribution error* [48], which has been shown for immigrants from Turkey and Italy in the German educational context [22].

A further education-related consequence of negative competence stereotypes is *stereotype threat* [49]. In the German context, negative competence-related stereotypes were shown to reduce Turkish-origin immigrants’ academic performance [22, 50, 51]. The current research showed that recently arrived immigrant groups from the Balkans and North Africa were stereotyped even more negatively on the competence dimension than the more established group of Turkish-origin immigrants in Germany. Therefore, it can be assumed that stereotype threat effects will also impede these groups’ integration into the German educational system [41]. Results of the current research are thus highly relevant for immigrant education and integration, because they show which immigrant groups are especially likely to experience adverse consequences of negative warmth and competence stereotypes.

#### Reducing negative stereotypes about immigrant groups

A prominent way to reduce negative stereotypes and prejudice is intergroup contact [29]. Positive contact between group members can–under specific conditions–improve intergroup relations and reduce negative stereotypes and prejudice [27]. One goal of applied social-psychological research to foster immigrant integration could therefore be to improve German receiving-society members’ stereotypes about immigrant groups. A pathway to more positive perceptions of immigrant groups is to create situations in which receiving-society members can engage in positive face-to-face or imagined contact with immigrants. For example, Brambilla and colleagues (2012) [52] showed that imagining positive contact with immigrant groups improved receiving-society members’ stereotypes about immigrant groups who were generally stereotyped as low on warmth and competence or as ambivalent in the Italian context. Brambilla and colleagues (2013) [53] showed that actual contact to immigrants not only improved stereotypes, but was also associated with more positive behavioral intentions towards immigrants in Italy. Furthermore, a recent study in the German context by Kotzur, Schäfer, and Wagner (2018) showed that positive contact with an asylum seeker increased German participants’ warmth perceptions, decreased the intergroup emotions of contempt and envy, and increased behavioral tendencies of solidarity-based collective action towards asylum seekers [28]. This means that interventions to create positive contact situations for Germans and immigrant groups are a promising route to increase or strengthen receiving-society members’ positive views of immigrants. However, research has also pointed to potential downsides of intergroup contact for the minority group, as positive contact with majority group members has been shown to reinforce system-justifying ideologies and thus decrease willingness to engage in collective action to change the status quo [54–56].

### Limitations and future directions

First, due to the cross-sectional nature of the current study, causal relations between variables could not be investigated. Longitudinal or experimental studies could be a fruitful agenda for future research. The assessment of socio-structural variables, stereotypes and behavioral tendencies at different time points would allow for testing causal relations. Variables might also be reciprocally related, forming a recursive cycle of stereotypes and integration. Longitudinal investigations could also shed light on how perceptions of immigrant groups develop over time. For example, immigrant groups who have recently migrated to Germany and were stereotyped as moderately warm and competent in the current study might receive more positive stereotypes over time [12].

Second, future studies should include recent developments in research on intergroup stereotypes. There is evidence that the warmth dimension is comprised of sociability (i.e., friendliness, likability) and morality (i.e., honesty, trustworthiness) [57]. Morality is relevant in the intergroup context as it predicts positive ingroup evaluations [58], information gathering [59] and outgroup impression formation [60]. Furthermore, morality mediated the effects of intergroup contact on more positive behavioral intentions towards immigrants in Italy [53]. Therefore, future studies should assess sociability and morality separately and investigate whether they are differentially related to behavioral tendencies towards various immigrant groups. The stereotype dimension of conservative-progressive beliefs [61] might also be relevant, especially for the perceptions of culturally distant immigrant groups. To create a more encompassing picture of socio-structural variables and cognitive, affective as well as behavioral aspects of intergroup bias towards a variety of immigrant groups in the German context, future studies should include emotional tendencies as measures of the affective component of bias in the path model (but see Kotzur, Schäfer, & Wagner (2018), who did this for the group of asylum seekers [28]).

Third, the hypotheses about stereotypes predicting majority integration efforts [8] as a further measure of active facilitation could not be tested in the current study, as the scale was adapted to reduce questionnaire length, but failed to show sufficient internal consistencies for some immigrant groups. Thus, future studies should include the complete scale for each immigrant group. Finally, although participants who likely speeded through the survey were excluded from the data analyses, we acknowledge that the study did not include an attention check to identify participants that may not have been paying attention or did not answer the survey seriously.

## Conclusion

In light of the societal challenges of immigrant integration in Germany, the current study underscores the important role of receiving-society members’ stereotypes for immigrant integration: Whether the social climate towards immigrant groups in Germany is welcoming or unwelcoming predicts stereotypes towards immigrant groups, which translate to behavioral intentions of support–likely leading to higher integration–or harm–likely leading to higher discrimination of immigrant groups. By investigating a broad selection of immigrant groups living in Germany, the current research contributes to the understanding that immigrants of different ethnic origins are not perceived alike, but that members of the (German) receiving society have differentiated social perceptions of immigrant groups.
